# Development of an RNA sequencing panel to detect gene fusions in thyroid cancer

**DOI:** 10.5808/gi.21061

**Published:** 2021-12-31

**Authors:** Dongmoung Kim, Seung-Hyun Jung, Yeun-Jun Chung

**Affiliations:** 1 Department of Biomedicine & Health Sciences, Graduate School, The Catholic University of Korea, Seoul 06591, Korea; 2 Department of Biochemistry, The Catholic University of Korea, Seoul 06591, Korea; 3 Precision Medicine Research Center, Integrated Research Center for Genome Polymorphism, College of Medicine, The Catholic University of Korea, Seoul 06591, Korea; 4 Department of Microbiology, College of Medicine, The Catholic University of Korea, Seoul 06591, Korea

**Keywords:** fusion, next-generation sequencing, RNA sequencing panel, thyroid cancer

## Abstract

In addition to mutations and copy number alterations, gene fusions are commonly identified in cancers. In thyroid cancer, fusions of important cancer-related genes have been commonly reported; however, extant panels do not cover all clinically important gene fusions. In this study, we aimed to develop a custom RNA-based sequencing panel to identify the key fusions in thyroid cancer. Our ThyChase panel was designed to detect 87 types of gene fusion. As quality control of RNA sequencing, five housekeeping genes were included in this panel. When we applied this panel for the analysis of fusions containing reference RNA (HD796), three expected fusions (*EML4-ALK*, *CCDC6-RET*, and *TPM3-NTRK1*) were successfully identified. We confirmed the fusion breakpoint sequences of the three fusions from HD796 by Sanger sequencing. Regarding the limit of detection, this panel could detect the target fusions from a tumor sample containing a 1% fusion-positive tumor cellular fraction. Taken together, our ThyChase panel would be useful to identify gene fusions in the clinical field.

## Introduction

Thyroid cancer is one of the most rapidly increasing cancers throughout the world, including South Korea [1]. Although most thyroid cancers show more favorable behavior than other cancers, and the 5-year disease-specific survival of thyroid cancer is above 98% [2], some thyroid cancers show aggressive behavior such as distant metastasis [3].

Next-generation sequencing (NGS) technology and efforts to identify genetic alterations in cancers, such as the Cancer Genome Atlas, have revealed genetic alteration profiles in diverse cancers [4]. In addition to mutations and copy number alterations, gene fusions are commonly identified in cancers, including thyroid cancer [5,6]. Gene fusions are mainly caused by chromosomal rearrangement; therefore, fusion events may have more tumorigenic implications than point mutations because cancer-related genes such as the *RET* oncogene can be overactivated through gene fusion [7]. The most common fusion events in thyroid cancer are the *RET*/*PTC* rearrangement in papillary thyroid cancer (10%–30%) and *PPARG-PAX8* rearrangement in follicular thyroid cancer (30%–60%) [8]. Various other gene fusions have also been identified in thyroid cancer, including *RET*, *THADA*, *NTRK1*, *NTRK3*, *ALK*, *BRAF*, *MET*, and *FGFR2* [8]. It is well known that gene fusions can affect the tumor behavior and prognosis of thyroid cancer [9,10]. For example, *NTRK1/3* fusions have been reported to be associated with advanced tumor stage and aggressive lymphovascular invasion [11-13]. Tumors with *ALK* fusions have been suggested to have a higher likelihood of dedifferentiation [14]. Therefore, detecting gene fusion events is essential both for diagnostic purposes and for predicting patients’ prognoses.

From a technical standpoint, the detection of fusion genes by DNA-based NGS is almost impossible due to the presence of diverse-sized intronic sequences between the fusion target exons. Therefore, RNA-based NGS panel analyses are commonly conducted to detect the target fusions in thyroid cancer in addition to the use of DNA-based NGS to detect somatic mutations. RNA-based NGS panels should include housekeeping genes. Since housekeeping genes are expressed in all tissue compartments and cell types, they can be used for quality control and normalization of NGS data [15]. In addition, housekeeping genes may drive the expression of fusion genes such as *VIT-ALK* in lung adenocarcinoma [16,17]. Multiple panels for thyroid molecular analysis have been developed [9]. Among them, ThyroSeq, a DNA- and RNA-based NGS assay including 112 genes, is the most commonly used panel across the world; it can detect more than 100 genetic alterations, including major gene mutations, fusions, and gene expression alterations [18]. This panel provides high accuracy for detecting all common types of thyroid cancer and parathyroid lesions using a fine-needle aspiration sample. However, no extant panels cover all clinically important gene fusions in thyroid cancer.

In this study, we aimed to develop a custom RNA-based NGS panel to identify the important fusion events in thyroid cancer. In addition to the key fusions in thyroid cancer, uncommonly reported fusions and fusion subtypes were also included in this panel.

## Methods

### Samples

In this study, we used two standard materials: HD796 (Horizon Discovery, Cambridge, UK) as a fusion-positive control and HD783 (Horizon Discovery) as a fusion-negative control. HD796 is a formalin-fixed paraffin-embedded (FFPE) tissue that contains the *EML4-ALK*, *CCDC6-RET*, *SLC34A2-ROS1*, *TPM3-NTRK1*, and *ETV6-NTRK3* fusions. HD783 is an FFPE sample that does not contain those fusions. RNA was extracted from the FFPE sample using an FFPE Total RNA Miniprep System kit (Promega, Madison, WI, USA). The quality and quantity of RNA samples were determined using a NanoDrop 2000c spectrophotometer (Thermo Fisher Scientific, Waltham, MA, USA).

### Library preparation

cDNAs were synthesized using a SuperScript VILO cDNA Synthesis Kit (Thermo Fisher Scientific) and used for NGS library preparation. The libraries were manually constructed using our custom thyroid fusion panel, ThyChase. The amplicon library was prepared with the Ion Plus Fragment Library Kit (Life Technologies, Waltham, MA, USA) and the Ion Xpress Barcode Adapters Kit (Life Technologies) according to the manufacturer's instructions. In detail, 10 μL of cDNA was amplified in reaction mixtures of 59 μL containing 45 μL of Platinum PCR SuperMix High Fidelity and 4 μL of ThyChase panel. Polymerase chain reaction (PCR) was performed with a GeneAmp 9700 thermal cycler (Thermo Fisher Scientific) under the conditions of 95°C for 2 min followed by 35 cycles of 95°C for 15 s, 58°C for 15 s, 68°C for 10 s, and a final hold at 4°C. Libraries were purified using 106 μL of AMPure XP Reagent (Beckman Coulter, Miami, FL, USA) on a magnetic stand (Thermo Fisher Scientific) and eluted with 25 μL of low tris-EDTA buffer. Then, adapter ligation and nick repairing were performed to make barcode sequencing adapters (Ion Xpress Barcode Adapters, Thermo Fisher Scientific). Finally, the libraries were quantified using quantitative PCR (qPCR; Ion Library Quantitation Kit, Thermo Fisher Scientific) on a QuantStudio 12K Flex Real-Time PCR System qPCR machine (Thermo Fisher Scientific).

### Template preparation and NGS reaction

Emulsion PCR, bead enrichment, and chip loading procedures were automatically performed on an Ion Chef instrument (Thermo Fisher Scientific) using Ion 510, 520, and 530 Kits (Thermo Fisher Scientific). A planned run was created for each chip within Ion Torrent Suite Software v5.12.1 (Thermo Fisher Scientific) with the template size set at 200 bp. The NGS libraries were then sequenced on an Ion S5 XL sequencer (Thermo Fisher Scientific) [18].

### Data analysis

Raw sequence data were analyzed with the Torrent Suite (version 5.12.1, Thermo Fisher Scientific). A custom reference genome was assembled to contain sequences of the 87 designed target fusions and five housekeeping genes based on hg19. To call the mapped sequence data, we used Torrent Coverage Analysis (version 5.12.0.0). More than five support reads were considered as fusion-positive. The identified fusions were then manually inspected in the Integrative Genomics Viewer (IGV, Broad Institute, Cambridge, MA, USA). The mean sequencing depth was 5,189× (range, 3,665× to 6,729×) across the entire target region (Supplementary Table 1). The dataset for the current study is available from the corresponding author upon reasonable request.

### Limit of fusion detection and validation

To determine the limit of detection (LOD) of fusions, we diluted the RNA extracted from the NCI-H2228 cell line (*EML4-ALK* fusion-positive) by mixing it with the RNA extracted from the FTC-133 cell line (*EML4-ALK* fusion-negative) from 100% to 0.5%. The RNAs were subjected to RNA sequencing using the ThyChase panel. To verify the fusions identified by the ThyChase panel, we performed Sanger sequencing of the fusion amplicons.

## Results and Discussion

### Design of the RNA sequencing panel

**Fig. 1.**
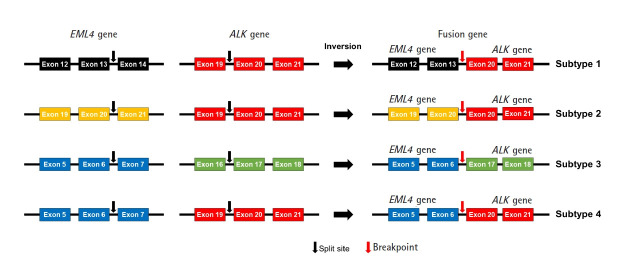
Subtypes of *EML4-ALK* fusion.

We designed an NGS panel named ThyChase containing 92 genes, targeting 87 gene fusion types and five housekeeping genes. The fusion targets were selected based on previous reports and the COSMIC database [7,8,19-64]. Of the 15 fusion targets, eight (*RET*, *THADA*, *BRAF*, *ALK*, *FGFR2*, *NTRK1*, *NTRK3*, and *PPARG*) are known to have multiple fusion partners, while the other seven are known to have a single fusion partner. Details of the fusion genes and their fusion partners are listed in Table 1. In some fusions, there are different fusion breakpoints, although the fusion partners are the same. For example, this panel can detect four fusion breakpoints of *EML4-ALK* fusion (exon 13 of *EML4* - exon 20 of *ALK*, exon 20 of *EML4* - exon 20 of *ALK*, exon 6 of *EML4* - exon 17 of *ALK*, and exon 6 of *EML4* - exon 20 of *ALK*) (Fig. 1). In total, 27 fusion subtypes can be discriminated with this panel (Supplementary Table 2). In addition to gene fusion, ThyChase includes five housekeeping genes for quality control of the experimental procedures of RNA sequencing and analysis: *CHMP2A*, *JUN*, *FBXW2*, *MET*, and *PUM1*.

### Validation and optimization of the panel

We used HD796 RNA as a fusion-positive standard material. HD796 contains *EML4-ALK*, *CCDC6-RET*, *TPM3-NTRK1*, *SLC34A2-ROS1*, and *ETV6-NTRK3* gene fusions [65]. Of the five fusions, three (*EML4-ALK*, *CCDC6-RET*, and *TPM3-NTRK1*) were included in our ThyChase panel. The *ETV6-NTRK3* gene fusion was also included in ThyChase; however, the *ETV6-NTRK3* fusion breakpoint of the HD796 RNA was different from the fusion breakpoint covered by ThyChase. Therefore, we targeted the three gene fusions for optimization and validation of ThyChase. HD783 RNA was used as a fusion-negative control that did not harbor any of the above-mentioned gene fusions [65].

**Fig. 2.**
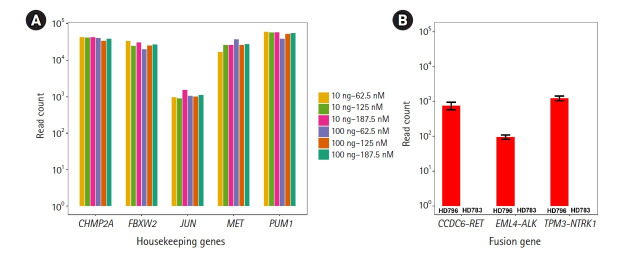
Technical validation of RNA sequencing and detection of gene fusion. (A) RNA expression levels of the five housekeeping genes. We applied six different amplification conditions: two different amounts of template RNA were applied (10 ng and 100 ng) with three different primer concentrations (62.5, 125, and 187.5 nM). These six combinations are represented as different colors in the plot. The X-axis represents the gene name; the Y-axis represents read counts. (B) Identification of the fusions (*EML4-ALK*, *CCDC6-RET*, and *TPM3-NTRK1*) from the HD796 and HD783 RNAs. The Y-axis represents read counts.

Before detecting the gene fusions, as technical validation, we checked whether the RNA expression of the housekeeping genes included in this panel could be stably detected under various experimental conditions with the HD796 RNA. As expected, expression of the housekeeping genes was stably detected and the read counts mapped to each target gene were above 10^2^, suggesting that our custom thyroid gene fusion panel is suitable for RNA sequencing (Fig. 2A). In parallel, to optimize the library preparation, we applied six different amplification conditions: two different amounts of template RNA were applied (10 ng and 100 ng) with three different primer concentrations (62.5, 125, and 187.5 nM). All five genes showed similar levels of the sequencing read counts in the six conditions (Fig. 2A). Therefore, we set the reaction condition as 10 ng of template RNA and 187.5 nM of primers. Regarding the quality control of ThaChase, we set >10^2^ read counts for every housekeeping gene as a threshold of a reliable RNA sequencing reaction, meaning that we could interpret the gene fusions identified by this panel sequencing analysis as true when the read counts of all housekeeping genes were above 10^2^.

### Detection of gene fusions

We next examined the three (*EML4-ALK*, *CCDC6-RET*, and *TPM3-NTRK1*) fusions in the HD796 reference RNA. RNA panel sequencing was performed based on the optimized reaction conditions described above. As expected, all three fusion targets were successfully detected in the HD796 RNA, whereas they were not identified in the HD783 RNA (Fig. 2B). When we checked the read count of the housekeeping genes, all the target genes had >10^2^ read counts (Supplementary Fig. 1), suggesting that the fusions detected by our thyroid gene fusion panel were reliable. These data also support the specificity of the fusions detected by our ThyChase panel.

**Fig. 3.**
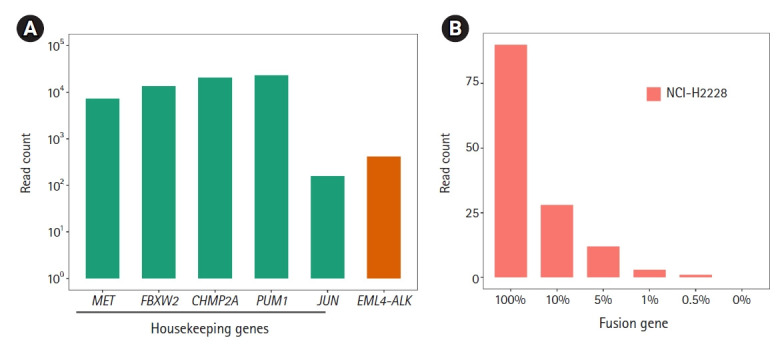
Identification of *EML4-ALK* fusion from the NCI-H2228 cell line and limits of detection (LOD). (A) Identification of the *EML4-ALK* fusion. All five housekeeping genes showed >10^2^ read counts. (B) To determine the LOD of the fusion, we diluted the NCI-H2228 RNA from 100% to 0.5% and performed RNA sequencing.

Next, we applied ThyChase to cell lines. For this, we used the RNA extracted from a lung cancer cell line (NCI-H2228), which is known to harbor the *EML4-ALK* fusion [66]. As expected, the *EML4-ALK* fusion was successfully detected in the NCI-H2228 cell line (Fig. 3A). Through this experiment, we confirmed that the ThyChase panel could identify the target fusions from cancer cell lines in addition to the fusion-positive reference RNA. This result suggests that our system is applicable for cancer samples.

To determine the LOD of the ThyChase panel for calling fusions with high confidence, the lowest tumor percent with high-confidence detection was examined. To achieve this, we analyzed NCI-H2228 (*EML4-ALK* fusion-positive) samples that were diluted with FTC-133 (*EML4-ALK* fusion-negative) by different dilution factors (100% to 0.5%). As a result, the read count of *EML4-ALK* fusion decreased in a dose-dependent manner from 100% to 0.5%, and we could identify fusion-supporting reads from the 0.5% fusion-positive tumor cellular fraction (Fig. 3B). However, the lowest percentage satisfying our high-confidence fusion calling criterion (>5 fusion-supporting reads) was a >1% fusion-positive tumor cellular fraction. Therefore, a 1% tumor fraction was determined to be the LOD of our assay.

### Verification of fusion breakpoints

**Fig. 4.**
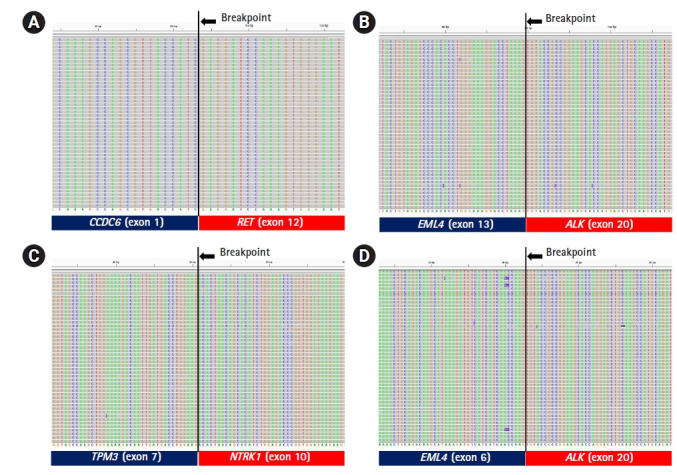
Integrative Genomics Viewer plot of the fusion breakpoints. (A) *CCDC6-RET* fusion breakpoint (exon 1 of *CCDC6* and exon 12 of *RET*). (B) *EML4-ALK* fusion breakpoint (exon 13 of *EML4* and exon 20 of *ALK*). (3) *TPM3-NTRK1* fusion breakpoint (exon 7 of *TPM3* and exon 10 of *NTRK1*). (4) *EML4-ALK* fusion breakpoint (exon 6 of *EML4* and exon 20 of *ALK*).

To verify the three fusions identified by our ThyChase panel from HD796 RNA, the RNA sequencing results were visualized using IGV. IGV showed the *CCDC6-RET* fusion breakpoint where exon 1 of *CCDC6* was fused with exon 12 of *RET* (Fig. 4A). In the *EML4-ALK* fusion, exon 13 of *EML4* was fused with exon 20 of *ALK* (Fig. 4B). In the breakpoint of *TPM3-NTRK1* fusion, exon 7 of *TPM3* and exon 10 of *NTRK1* were fused (Fig. 4C). In addition, we confirmed the fusion subtype of *EML4-ALK* fusion, which was identified from NCI-H2228, where exon 6 of *EML4* was fused with exon 20 of *ALK* (Fig. 4D).

**Fig. 5.**
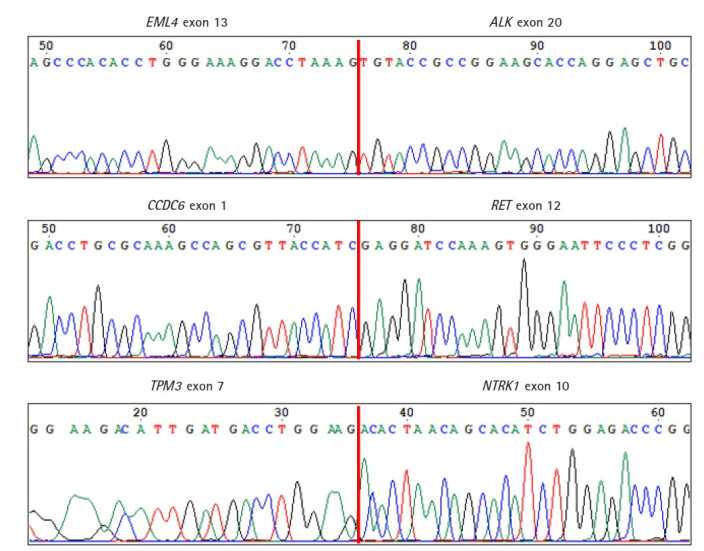
Confirmation of the fusion breakpoints by Sanger sequencing. Vertical red lines represent fusion breakpoints.

As a final confirmation of the fusions and their breakpoints, we performed Sanger sequencing of the amplicons of the fusions from HD796. Sanger sequencing revealed the fusion breakpoint sequences of the *EML4-ALK* (exon 13 of *EML4* and exon 20 of *ALK*), *CCDC6-RET* (exon 1 of *CCDC6* and exon 12 of *RET*), and *TPM3-NTRK1* (exon 7 of *TPM3* and exon 10 of *NTRK1*) fusions (Fig. 5).

In conclusion, we developed an RNA-based sequencing panel focused on identifying fusions in thyroid cancer. The ThyChase panel was designed to detect 87 gene fusion types. As quality control for RNA sequencing, five housekeeping genes were included in this panel. When we applied this panel for the analysis of fusions contained in the reference RNA (HD796), the three expected fusions (*EML4-ALK*, *CCDC6-RET*, and *TPM3-NTRK1*) were successfully identified. We also confirmed that this fusion-focused panel could identify the target fusions from a cancer cell line in addition to the fusion-positive reference RNA. In terms of the LOD, this panel could detect the target fusions from a tumor sample containing a 1% fusion-positive tumor cellular fraction. We finally verified the fusion breakpoint sequences of the three fusions from HD796. Although we could not verify all of the designed fusions in this study due to limitations of the fusion reference materials, all the data in this study indicate that the ThyChase panel can reliably identify the key fusions in thyroid cancer. Taken together, the ThyChase panel would be useful to identify gene fusions in the clinical field.

## Figures and Tables

**Table 1.** The fusion genes and their fusion partners contained in the panel

Fusion gene	Partner gene(s)	Reference
*THADA*	*IGF2BP3, LOC389473, LOC100505678, TRA2A*	[5,19,46,47,54]
*RET*	*CCDC6, ERC1, FKBP15, GOLGA5, HOOK3, KIAA1217, KTN1, NCOA4, PCM1, PRKAR1A, TRIM24, TRIM33, TRIM27, SPECC1L, TBL1XR1, AKAP13, DLG5, SQSTM1, CCDC186, AFAP1L2, PPFIBP2, KIF5B,*	[7,20-39,44,46,54]
*BRAF*	*AKAP9, AGK, LMO7, BCL2L11, CCNY, FAM114A2, OSBPL1A, OSBPL9, MACF1, POR, SND1, MKRN1, ZC3HAV1, PICALM, NFYA, AP3B1*	[23,40-47,54]
*ALK*	*STRN, EML4, GFPT1, GTF2IRD1, CCDC149,*	[23,48-53]
*FGFR2*	*WARS, KIAA1598, OFD1, VCL*	[46,47,59]
*NTRK1*	*IRF2BP2, TFG, TPM3, TPR, SQSTM1, SSBP2*	[46,47,54-58]
*NTRK3*	*ETV6, RBPMS, SQSTM1, EML4*	[41,47,54,60]
*PPARG*	*CREB3L2, PAX8*	[8,41,61,62]
*UACA*	*LTK*	[47]
*MET*	*TFG*	[23]
*SS18*	*SLC5A11*	[63]
*RNF213*	*SLC26A11*	[46]
*ROS1*	*CCDC30*	[64]
*RAF1*	*AGGF1*	[23]
*EZR*	*ERBB4*	[46]
