# Supplementary material for: Development of an RNA sequencing panel to detect gene fusions in thyroid cancer
Source: Genomics Inform. 2021 Dec 31;19(4):e41. doi: 10.5808/gi.21061 (PMC8752984; doi:10.5808/gi.21061)
Supplement: Supplemental Fig. 1. [file gi-21061suppl1.pdf]

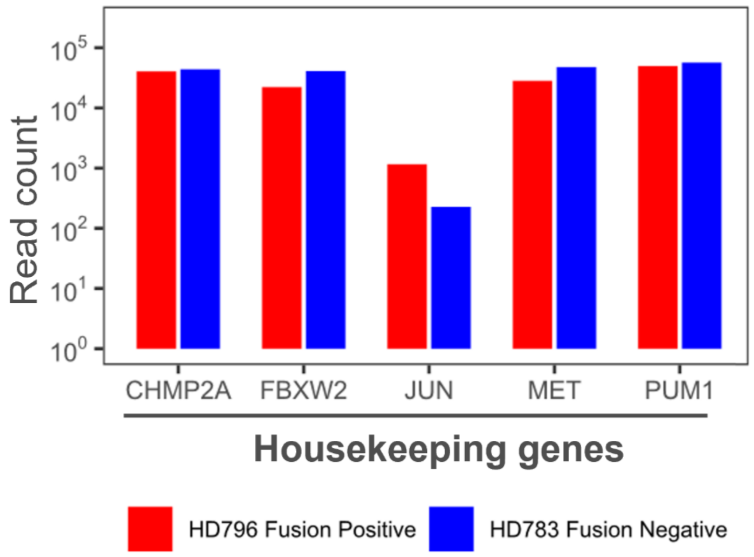

Supplementary Fig. 1. Read count of housekeeping genes. RNA sequencing of both HD796 and HD783 showed >10^2 read counts for all five housekeeping genes.

References

1. Panebianco F, Kelly LM, Liu P, Zhong S, Dacic S, Wang X, et al. THADA fusion is a mechanism of IGF2BP3 activation and IGF1R signaling in thyroid cancer. Proc Natl Acad Sci U S A 2017;114:2307-2312.

2. Liang J, Cai W, Feng D, Teng H, Mao F, Jiang Y, et al. Genetic landscape of papillary thyroid carcinoma in the Chinese population. J Pathol 2018;244:215-226.

3. Di Cristofaro J, Marcy M, Vasko V, Sebag F, Fakhry N, Wynford-Thomas D, et al. Molecular genetic study comparing follicular variant versus classic papillary thyroid carcinomas: association of N-ras mutation in codon 61 with follicular variant. Hum Pathol 2006;37:824-830.

4. Santarpia L, Myers JN, Sherman SI, Trimarchi F, Clayman GL, El-Naggar AK. Genetic alterations in the RAS/RAF/mitogen-activated protein kinase and phosphatidylinositol 3-kinase/Akt signaling pathways in the follicular variant of papillary thyroid carcinoma. Cancer 2010;116:2974-2983.

5. Sheils OM, O'Leary JJ, Sweeney EC. Assessment of ret/PTC-1 rearrangements in neoplastic thyroid tissue using TaqMan RT-PCR. J Pathol 2000;192:32-36.

6. Stransky N, Cerami E, Schalm S, Kim JL, Lengauer C. The landscape of kinase fusions in cancer. Nat Commun 2014;5:4846.

7. Giannini R, Salvatore G, Monaco C, Sferratore F, Pollina L, Pacini F, et al. Identification of a novel subtype of H4-RET rearrangement in a thyroid papillary carcinoma and lymph node metastasis. Int J Oncol 2000;16:485-489.

8. Musholt PB, Imkamp F, von Wasielewski R, Schmid KW, Musholt TJ. RET rearrangements in archival oxyphilic thyroid tumors: new insights in tumorigenesis and classification of Hurthle cell carcinomas? Surgery 2003;134:881-889.

9. Elisei R, Romei C, Soldatenko PP, Cosci B, Vorontsova T, Vivaldi A, et al. New breakpoints in both the H4 and RET genes create a variant of PTC-1 in a post-Chernobyl papillary thyroid carcinoma. Clin Endocrinol (Oxf) 2000;53:131-136.

10. Sheu SY, Schwertheim S, Worm K, Grabellus F, Schmid KW. Diffuse sclerosing variant of papillary thyroid carcinoma: lack of BRAF mutation but occurrence of RET/PTC rearrangements. Mod Pathol 2007;20:779-787.

11. Liu S, Gao A, Zhang B, Zhang Z, Zhao Y, Chen P, et al. Assessment of molecular testing in fine-needle aspiration biopsy samples: an experience in a Chinese population. Exp Mol Pathol 2014;97:292-297.

12. Yoo SK, Lee S, Kim SJ, Jee HG, Kim BA, Cho H, et al. Comprehensive analysis of the transcriptional and mutational landscape of follicular and papillary thyroid cancers. PLoS Genet 2016;12:e1006239.

13. Hu X, Wang Q, Tang M, Barthel F, Amin S, Yoshihara K, et al. TumorFusions: an integrative resource for cancer-associated transcript fusions. Nucleic Acids Res 2018;46:D1144-D1149.

14. Hamatani K, Mukai M, Takahashi K, Hayashi Y, Nakachi K, Kusunoki Y. Rearranged anaplastic lymphoma kinase (ALK) gene in adult-onset papillary thyroid cancer amongst atomic bomb survivors. Thyroid 2012;22:1153-1159.

15. Kelly LM, Barila G, Liu P, Evdokimova VN, Trivedi S, Panebianco F, et al. Identification of the transforming STRN-ALK fusion as a potential therapeutic target in the aggressive forms of thyroid cancer. Proc Natl Acad Sci U S A 2014;111:4233-4238.

16. Zeng Q, Gao H, Zhang L, Qin S, Gu Y, Chen Q. Coexistence of a secondary STRN-ALK, EML4-ALK double-fusion variant in a lung adenocarcinoma patient with EGFR mutation: a case report. Anticancer Drugs 2021;32:890-893.

17. Greco A, Miranda C, Pagliardini S, Fusetti L, Bongarzone I, Pierotti MA. Chromosome 1 rearrangements involving the genes TPR and NTRK1 produce structurally different thyroid-specific TRK oncogenes. Genes Chromosomes Cancer 1997;19:112-123.

18. Greco A, Pierotti MA, Bongarzone I, Pagliardini S, Lanzi C, Della Porta G. TRK-T1 is a novel oncogene formed by the fusion of TPR and TRK genes in human papillary thyroid carcinomas. Oncogene 1992;7:237-242.

19. Leeman-Neill RJ, Kelly LM, Liu P, Brenner AV, Little MP, Bogdanova TI, et al. ETV6-NTRK3 is a common chromosomal rearrangement in radiation-associated thyroid cancer. Cancer 2014;120:799-807.

20. Ricarte-Filho JC, Li S, Garcia-Rendueles ME, Montero-Conde C, Voza F, Knauf JA, et al. Identification of kinase fusion oncogenes in post-Chernobyl radiation-induced thyroid cancers. J Clin Invest 2013;123:4935-4944.

21. Nikiforova MN, Biddinger PW, Caudill CM, Kroll TG, Nikiforov YE. PAX8-PPARgamma rearrangement in thyroid tumors: RT-PCR and immunohistochemical analyses. Am J Surg Pathol 2002;26:1016-1023.

22. Chia WK, Sharifah NA, Reena RM, Zubaidah Z, Clarence-Ko CH, Rohaizak M, et al. Fluorescence in situ hybridization analysis using PAX8- and PPARG-specific probes reveals the presence of PAX8-PPARG translocation and 3p25 aneusomy in follicular thyroid neoplasms. Cancer Genet Cytogenet 2010;196:7-13.
