# Supplementary material for: Development of an RNA sequencing panel to detect gene fusions in thyroid cancer
Source: Genomics Inform. 2021 Dec 31;19(4):e41. doi: 10.5808/gi.21061 (PMC8752984; doi:10.5808/gi.21061)
Supplement: Supplemental Table 1. [file gi-21061suppl2.pdf]

Supplementary Table 1. The description of ThyChase panel sequencing data

Sample name	Total aligned base reads	Total base reads on target	Bases in target regions	Percent base reads on target	Average base coverage depth	Uniformity of base coverage (%)	Target base coverage at 1× (%)	Target base coverage at 20× (%)	Target base coverage at 100× (%)	Target base coverage at 500× (%)
HD 796, 10 ng, 62.5 nM	121,264,367	85,453,298	14,145	70.47	6,041	18.51	50.74	37.14	26.00	22.23
HD 796, 10 ng,125 nM	114,295,605	80,918,260	14,145	70.80	5,721	19.43	50.80	38.70	26.93	23.17
HD 796, 10 ng, 187.5 nM	134,082,024	95,188,483	14,145	70.99	6,729	20.18	58.84	38.97	28.65	23.33
HD 796, 100 ng, 62.5 nM	140,797,088	59,339,599	14,145	42.15	4,195	16.54	48.12	28.72	23.03	19.25
HD796, 100 ng, 125 nM	159,108,023	67,043,955	14,145	42.14	4,740	19.84	53.50	35.00	25.17	22.96
HD 796, 100 ng, 187.5 nM	182,685,733	78,580,456	14,145	43.01	5,555	19.03	56.43	36.06	25.05	22.96
HD 796, fusion positive	97,489,124	68,815,354	14,145	70.59	4,865	17.04	54.22	31.14	24.76	22.18
HD783, fusion negative	73,728,581	51,843,612	14,145	70.32	3,665	17.62	47.35	30.32	22.35	18.72
