# Supplementary material for: Development of an RNA sequencing panel to detect gene fusions in thyroid cancer
Source: Genomics Inform. 2021 Dec 31;19(4):e41. doi: 10.5808/gi.21061 (PMC8752984; doi:10.5808/gi.21061)
Supplement: Supplemental Table 2. [file gi-21061suppl3.pdf]

Supplementary Table 2. Twenty-seven kinds of fusion subtypes

Fusion genes (# Subtype)	Subtypes defined by specific gene fusions	Reference
THADA-IGF2BP3 (4)	*THADA* (exon 30) – *IGF2BP3* (exon 3) *THADA* (exon 31) – *IGF2BP3* (exon 2) *THADA* (exon 36) – *IGF2BP3* (exon 3) *THADA* (exon 36) – *IGF2BP3* (exon 2)	[1,2]
THADA-LOC389473 (2)	*THADA* (exon 28) – *LOC389473 THADA* (exon 29) – *LOC389473*	[1]
CCDC6-RET (3)	*CCDC6* (exon 1) – *RET* (exon 12) *CCDC6* (exon 2) – *RET* (exon 12) *CCDC6* (exon 8) – *RET* (exon 11)	[3-9]
NCOA4-RET (2)	*NCOA4* (exon 8) – *RET* (exon 12)<br>*NCOA4* (exon 7) – *RET* (exon 12)	[10-12]
SND1-BRAF (2)	*SND1* (exon 10) – *BRAF* (exon 9)<br>*SND1* (exon 18) – *BRAF* (exon 10)	[12,13]
EML4-ALK (4)	*EML4* (exon 13) – *ALK* (exon 20) *EML4* (exon 20) – *ALK* (exon 20) *EML4* (exon 6) – *ALK* (exon 17) *EML4* (exon 6) – *ALK* (exon 20)	[6,14-16]
TPR-NTRK1 (2)	*TPR* (exon 21) – *NTRK1* (exon 10)<br>*TPR* (exon 6) – *NTRK1* (exon 12)	[17,18]
SQSTM1-NTRK1 (2)	*SQSTM1* (exon 6) – *NTRK1* (exon 10)<br>*SQSTM1* (exon 6) – *NTRK1* (exon 12)	[12,13]
ETV6-NTRK3 (2)	*ETV6* (exon 4) – *NTRK3* (exon 14)<br>*ETV6* (exon 5) – *NTRK3* (exon 14)	[19,20]
PAX8-PPARG (4)	*PAX8* (exon 10) – *PPARG* (exon 2) *PAX8* (exon 8) – *PPARG* (exon 2) *PAX8* (exon 9) – *PPARG* (exon 2) *PAX8* (exon 7) – *PPARG* (exon 2)	[21,22]
